# Quantification of tumour vasculature and hypoxia by immunohistochemical staining and HbO_2_ saturation measurements

**DOI:** 10.1038/sj.bjc.6690072

**Published:** 1999-02

**Authors:** B M Fenton, S F Paoni, J Lee, C J Koch, E M Lord

**Affiliations:** Department of Radiation Oncology, University of Rochester School of Medicine, Rochester, NY, USA; Department of Microbiology and Immunology, University of Rochester School of Medicine, Rochester, NY, USA; Department of Radiation Oncology, University of Pennsylvania, Philadelphia, PA, USA

**Keywords:** image analysis, angiogenesis, blood vessels, tumour oxygenation, oxyhaemoglobin, hypoxic marker

## Abstract

Despite the possibility that tumour hypoxia may limit radiotherapeutic response, the underlying mechanisms remain poorly understood. A new methodology has been developed in which information from several sophisticated techniques is combined and analysed at a microregional level. First, tumour oxygen availability is spatially defined by measuring intravascular blood oxygen saturations (HbO_2_) cryospectrophotometrically in frozen tumour blocks. Second, hypoxic development is quantified in adjacent sections using immunohistochemical detection of a fluorescently conjugated monoclonal antibody (ELK3-51) to a nitroheterocyclic hypoxia marker (EF5), thereby providing information relating to both the oxygen consumption rates and the effective oxygen diffusion distances. Third, a combination of fluorescent (Hoechst 33342 or DiOC_7_(3)) and immunohistological (PECAM-1/CD31) stains is used to define the anatomical vascular densities and the fraction of blood vessels containing flow. Using a computer-interfaced microscope stage, image analysis software and a 3-CCD colour video camera, multiple images are digitized, combined to form a photo-montage and revisited after each of the three staining protocols. By applying image registration techniques, the spatial distribution of HbO_2_ saturations is matched to corresponding hypoxic marker intensities in adjacent sections. This permits vascular configuration to be related to oxygen availability and allows the hypoxic marker intensities to be quantitated in situ. © 1999 Cancer Research Campaign


*British Journal of Cancer* (1999) **79**(3/4), 464–471
© 1999 Cancer Research Campaign
Article no. bjoc.1998.0072

### Quantification of tumour vasculature and hypoxia by immunohistochemical staining and $HbO_2$ saturation measurements

**BM Fenton[1], SF Paoni[1], J Lee[2], CJ Koch[3] and EM Lord[2]**

[1]Department of Radiation Oncology, University of Rochester School of Medicine, Rochester, NY, USA; [2]Department of Microbiology and Immunology, University of Rochester School of Medicine; [3]Department of Radiation Oncology, University of Pennsylvania, Philadelphia, PA, USA

**Summary** Despite the possibility that tumour hypoxia may limit radiotherapeutic response, the underlying mechanisms remain poorly understood. A new methodology has been developed in which information from several sophisticated techniques is combined and analysed at a microregional level. First, tumour oxygen availability is spatially defined by measuring intravascular blood oxygen saturations ($HbO_2$) cryospectrophotometrically in frozen tumour blocks. Second, hypoxic development is quantified in adjacent sections using immunohistochemical detection of a fluorescently conjugated monoclonal antibody (ELK3-51) to a nitroheterocyclic hypoxia marker (EF5), thereby providing information relating to both the oxygen consumption rates and the effective oxygen diffusion distances. Third, a combination of fluorescent (Hoechst 33342 or $DiOC_7(3)$) and immunohistological (PECAM-1/CD31) stains is used to define the anatomical vascular densities and the fraction of blood vessels containing flow. Using a computer-interfaced microscope stage, image analysis software and a 3-CCD colour video camera, multiple images are digitized, combined to form a photo-montage and revisited after each of the three staining protocols. By applying image registration techniques, the spatial distribution of $HbO_2$ saturations is matched to corresponding hypoxic marker intensities in adjacent sections. This permits vascular configuration to be related to oxygen availability and allows the hypoxic marker intensities to be quantitated in situ.

**Keywords:** image analysis; angiogenesis; blood vessels; tumour oxygenation; oxyhaemoglobin; hypoxic marker

Tumour oxygenation has been shown to play an important role in radiotherapeutic response both in experimental tumours and in the clinic. To eliminate microregions of hypoxia, i.e. low oxygenation, numerous strategies have been used, generally aiming either to increase oxygen delivery to the tumour (Horsman et al, 1994) or to directly target the hypoxic cells using radiosensitizers or hypoxic cell cytotoxic agents (Brown and Giaccia, 1994). Several recent reports have also suggested the use of angiogenesis inhibitors (Klauber et al, 1997; O'Reilly et al, 1997) or thrombocytic agents (Huang et al, 1997) to directly target the tumour vasculature. A major shortcoming in evaluating the use of any of these approaches, however, is the inability to adequately quantify and understand the accompanying changes in tumour physiology.

The current study presents a method for combining several sophisticated techniques to obtain a comprehensive two-dimensional mapping of the relationships among tumour vascular configuration, oxygen transport and hypoxic development. First, tumour oxygen availability is spatially defined by measuring intravascular blood oxygen saturations ($HbO_2$) cryospectrophotometrically in a frozen tumour block. Second, hypoxic development, in relation to the intravascular oxygen availability, is quantified in an adjacent histological section, using immunohistochemical detection of a recently developed nitroheterocyclic hypoxia marker (EF5). Third, a combination of fluorescent and immunohistological stains is used to define the distribution of distances from the viable tumour cells to the nearest anatomical or perfused blood vessel.

*Received 1 October 1997*
*Revised 10 March 1998*
*Accepted 13 March 1998*

*Correspondence to:* BM Fenton, Box 704, University of Rochester Medical Center, Rochester, New York, 14642, USA

### MATERIALS AND METHODS

### Mice and tumour models

The KHT tumour, a sarcoma maintained in vivo, was passaged approximately every 2 weeks by i.m. inoculation of single-cell suspensions prepared by a mechanical dissociation procedure (Thomson and Rauth, 1974). Using 6- to 8-week-old female C3H/HeJ mice (Jackson Laboratories, Bar Harbor, ME, USA), $2 \times 10^5$KHT cells were inoculated i.m. into the hind limbs. Tumours were selected for analysis when they reached volumes of between 320 and 1100 $mm^3$ (as measured by callipers (volume = $\pi \bullet$diameter$^3$/6). Guidelines for the humane treatment of animals were followed as approved by the University Committee on Animal Resources.

### Injection of fluorescent stains and EF5 hypoxic marker

Localized areas of tumour hypoxia were assessed in frozen tissue sections by immunohistochemical identification of sites of 2-nitroimidazole metabolism. A pentafluorinated derivative (EF5) of etanidazole (synthesized by Dr R Vishnuvajjala of the National Cancer Institute) was injected i.v., 1 h before tumour freezing (0.2 ml of 10 mM EF5). Protein conjugates of EF5 have been used previously to immunize mice from which monoclonal antibodies were developed (Lord et al, 1993). These antibodies are extremely specific for the EF5 drug adducts that form when the drug is incorporated by hypoxic cells, and one of these, ELK3-51, has been well characterized. Regions of high EF5 metabolism in multicellular tumour spheroids, as well as murine and rat tumours, were visualized immunochemically using a fluorochrome (Cy3, Amersham) conjugated to the ELK3-51 antibody. To visualize blood vessels open to flow, either of two intravascular injected stains was used. The bis-benzamide Hoechst 33342 (H33342, Molecular Probes, Eugene, OR, USA) was injected into the tail vein 1 min before tumour freezing, at a concentration of 7.5 mg $kg^{-1}$ in saline (0.05 ml). This DNA-binding stain emits blue fluorescence and preferentially stains tumour cells adjacent to blood vessels (Olive and Durand, 1987; Trotter et al, 1989*a*). H33342 is removed very rapidly from the circulation (half-life of 2 minutes) and has been shown to be very stable once bound to DNA (Smith et al, 1988). As an alternative, $DiOC_7(3)$ (Molecular Probes) was also tested in a second group of tumours. Injections were administered i.v. 1 min before tumour freezing at a concentration of 1.0 mg $kg^{-1}$ (dissolved in 75% dimethyl sulphoxide in phosphate-buffered saline). This dose has been shown to provide optimal visualization of tumour vasculature by preferentially staining cells immediately adjacent to blood vessels (Trotter et al, 1989*b*). $DiOC_7(3)$ is removed very rapidly from the circulation (half-life of 3 min) and emits green fluorescence when excited by blue light (450 to 490-nm excitation, 510-nm dichroic, and 520-nm barrier filter). To eliminate potential artifacts related to the possible influence of the fluorescent stains on $HbO_2$ measurements, as well as the added stress due to the i.v. injections, separate tumour groups were used to: (1) relate EF5 uptake to corresponding intravascular $HbO_2$ determinations and (2) relate EF5 uptake to the various vascular markers.

### Tumour freezing and sectioning

To accelerate the freezing procedures and ensure that $HbO_2$ saturations were unchanged during freezing, tumours were first shaved, and a depilatory agent was applied. At appropriate times after treatment, the mice were cervically dislocated, and the tumours were immediately quick-frozen using a liquid nitrogen-cooled copper block and stored in cryotanks for later cryospectrophotometric analysis. Tumours for the $HbO_2$ analysis were sectioned using a strictly observed protocol: (1) tumours were first cut to approximately 5-mm cubes under liquid nitrogen, using a chisel; (2) tumours were then transferred to a –20°C cryostat and allowed to equilibrate under a liquid nitrogen-cooled stream of nitrogen gas for 10 min; (3) 70–80 14-µm thin sections were quickly cut and discarded, to reach a location nearer the tumour centre; (4) a jig holding four pins was used to pierce four defined reference holes into the tumour block (encompassing a roughly 4×5 mm area on one face of the tumour cube); (5) section thickness was set to 4.0-µm and another ten sections were quickly sliced (and discarded) under the cooled nitrogen gas, to prevent oxygen uptake in the tumour blood vessels during the sectioning; (6) the final two 4.0-µm sections (one for the immunohistochemistry control) were cut and mounted on poly-L-lysine-coated glass slides for later staining and imaging; and (7) the tumour block and cryostat mounting chuck was quickly removed and returned to the liquid nitrogen. Glass slides were stored at –20°C until imaged and stained. Tumour blocks could be stored indefinitely in liquid nitrogen before performing the cryospectrophotometric analysis.

### Cryospectrophotometric determination of intravascular $HbO_2$ saturations

For $HbO_2$ determinations, the exposed tumour surfaces were analysed on a liquid nitrogen-cooled microscope stage. Approximately 150 blood vessels were quantified per tumour, using green light to enhance visual contrast of the red blood cells, and selecting all vessels of diameter ≥ 8 µm (determined using an eyepiece reticle) that were located within the imaged region (see below). Spatial positions of the blood vessels (and reference pinholes) were recorded using stage micrometers, and intravascular $HbO_2$ saturations were determined cryospectrophotometrically, as previously described in detail (Fenton and Gayeski, 1990). Briefly, $HbO_2$ saturations were calibrated as a function of reflected light intensity at three discrete wavelengths, based on the spectral differences between oxy- and deoxy-haemoglobin. This means that only vessels containing red blood cells could be quantified, and all haemoglobin in a given vessel was averaged to obtain the final per cent oxyhaemoglobin. As optical density also varies as a function of haemoglobin concentration and light pathlength, the intensities at the three wavelengths were combined to normalize the measurement and to cancel out these dependencies, thus allowing vessels of widely varying haematocrit to be analysed using a single calibration curve. $HbO_2$ saturations were stored on disk with corresponding X and Y spatial coordinates.

### Immunohistochemistry

Immediately after cryostat sectioning, tumour slices were imaged for either H33342 or $DiOC_7(3)$ staining (as detailed below). Sections were then stored at –20°C (for up to 3 days, if necessary), before beginning the PECAM-1 [platelet endothelial cell adhesion molecule (Vecchi et al, 1994), also referred to as CD31] and EF5 hypoxic marker staining. On day 1 of this staining procedure, sections were fixed in cold 4% paraformaldehyde for 1 h, washed in phosphate-buffered saline (PBS), blocked in 5% normal mouse serum, washed in PBS and incubated with a 1:100 dilution of rat anti-mouse CD31 monoclonal antibody IgG2a,κ (MEC 13.3, PharMingen, San Diego, CA, USA) overnight at 4°C in a humid box. Slides were brought to room temperature at the start of day two, rinsed in a series of PBS washes, quenched in 3% hydrogen peroxide and incubated with a 1:25 dilution of peroxidase-conjugated mouse anti-rat IgG, (H+L) (Jackson Immunoresearch Laboratories, West Grove, PA, USA) for 1 h at room temperature in a humid box. After a PBS wash, sections were incubated with the chromagen AEC (3-amino-9-ethylcarbazole, AEC Substrate Kit for Horseradish Peroxidase, Vector Laboratories, Burlingame, CA, USA) for 30 min, at room temperature and in the dark. All remaining staining and imaging steps were also performed in the dark to minimize the loss of light-sensitive AEC staining intensity. After AEC incubation, sections were washed in a series of distilled water and PBS, blocked with 5% normal mouse serum and incubated with 100 µl of ELK3-51/Cy3 anti-EF5 antibody at 75 µg $ml^{-1}$ overnight at 4°C in a humid box. On day 3, sections were washed three times in PBS and fixed again in 1% paraformaldehyde for at least 10 min. Sections were then cover-slipped with PBS and imaged. Each tumour section had a corresponding control, incubated with buffer in place of antibodies and stained with AEC, to account for non-specific staining.

### Imaging equipment and procedures

The stained sections were imaged using an epi-fluorescence equipped Nikon microscope with 100 W mercury lamp illumination (10×/NA 0.3 and 20×/NA 0.5 objectives), digitized (FlashPoint frame grabber and Hitachi 3-CCD HV-C20 colour

© Cancer Research Campaign 1999

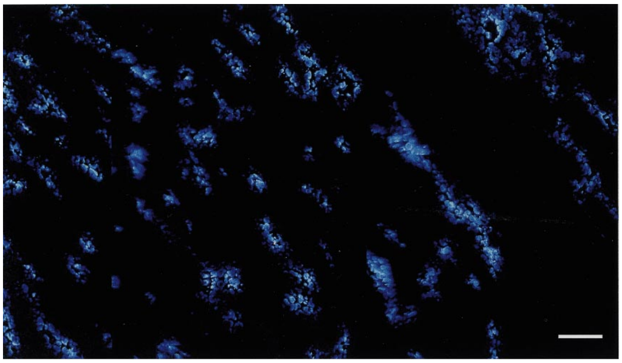

**Figure 1** An enlarged portion of the Hoechst 33342-stained montage. Vessels open to flow at the time of tumour freezing are identified by brightly blue stained cells around the vessel lumens. Bar = 100 µm

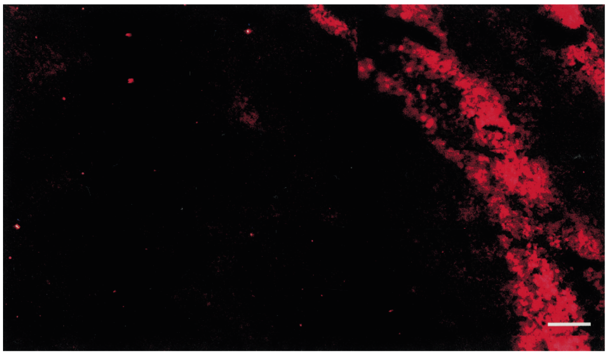

**Figure 2** EF5/Cy3-stained section corresponding to the area shown in Figure 1. The most hypoxic regions are identified by the most brightly stained red regions at the right, and correspond to the regions of Figure 1 in which no open blood vessels are located. Bar = 100 µm

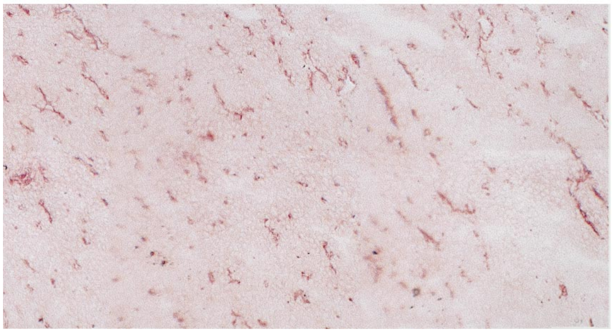

**Figure 3** PECAM-1/CD31-stained section corresponding to the areas shown in Figures 1 and 2. This stain preferentially marks endothelial cells and therefore highlights all blood vessels, whether open or closed to flow. Although blood vessels are present in the hypoxic regions highlighted in Figure 2, these vessels are generally not open to flow. Bar = 100 µm

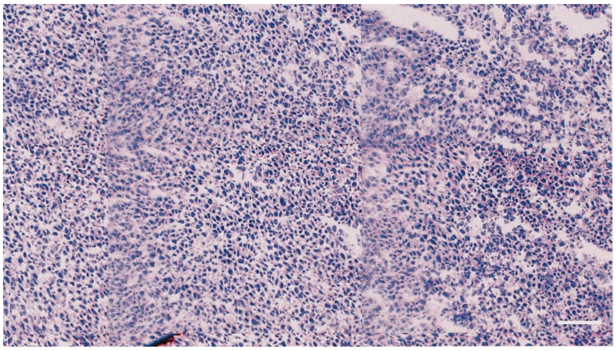

**Figure 4** Haematoxylin and eosin-stained section corresponding to Figures 1–3. This slide illustrates that the increased EF5 staining in Figure 2 does not correspond to regions of necrosis. Bar = 100 µm video camera), background-corrected and image-analysed using Image Pro software (Version 3.0, Media Cybernetics, Silver Spring, MD, USA) and a 133-MHz Pentium computer (96 Mb RAM). Colour images from adjacent microscope fields were automatically acquired and digitally combined (using a customized Image-Pro macro) to form 4×4 montages of the tumour cross-section (using the Image Pro Stage-Pro module, in conjunction with a Maertzhauser motorized stage and a Prior controller for the stage stepping motors). Single image frames were 640×480 pixels (corresponding to 579×434 µm on the histological slide, for the 20× objective), and 4×4 montages were 2520×1880 pixels (corresponding to 2.28×1.70 mm). For the 10×EF5 images, 6×6 montages corresponded to 6.94×5.18 mm.

Each section was scanned under each of four different staining conditions. First, epi-illumination images of the fluorescent blue H33342 staining (4×4 montages at 20×) were obtained immediately after the 4.0-µm sections were sliced on the cryostat (Figure 1). UV excitation was at 330–380 nm, with a 400-nm dichroic and a 420-nm barrier filter (UV-2A filter cube), and care was taken to avoid both normal tissue and sectioning artifacts. On day 3, after the immunohistochemical staining procedures were completed, the same tumour section used for the H33342 imaging was returned to the microscope stage and automatically rescanned using the same coordinates as for the initial 4×4 montages. Under epi-illumination (TRITC filter cube: 530- to 550-nm excitation, 565-nm dichroic and 580-nm barrier filter), fluorescent red-orange 4×4 (20×) montages were obtained showing the distribution of the Cy3 hypoxia staining (Figure 2). Next, using transmitted light (100 W Hg), matching brownish-red montages of the PECAM-1/CD31 staining were acquired (Figure 3). All fluorescent images of the EF5 hypoxic marker were obtained before the use of transmitted light for imaging of PECAM-1/CD31 staining, to prevent fading of the EF5 staining. Although AEC is light sensitive, the use of transmitted light does not decrease AEC staining intensity during the time required to obtain images of PECAM-1/CD31 staining. Finally, matched haematoxylin and eosin-stained images were taken to visualize cellular structures and necrosis (Figure 4).

### RESULTS

### Image processing

#### *PECAM-1 image enhancement*

The PECAM-1 image (anatomical blood vessels) was enhanced by first using the Image Pro 'colour segmentation' to identify blood vessels marked with the AEC stain. Specific 3×3 pixel colour samples were interactively selected and accumulated to obtain optimal discrimination between vessels and stroma (as determined visually), and a binary image of the selected colours was created. For automated counting of the vascular structures, the binary image was first subjected to a filtering operation (an 11×11 binary closing to fill in the gaps between contiguous endothelial cells of the same blood vessel) as shown in Figure 5. Here, the enhanced blood vessels are superimposed over the EF5/Cy3 staining to

 © Cancer Research Campaign 1999

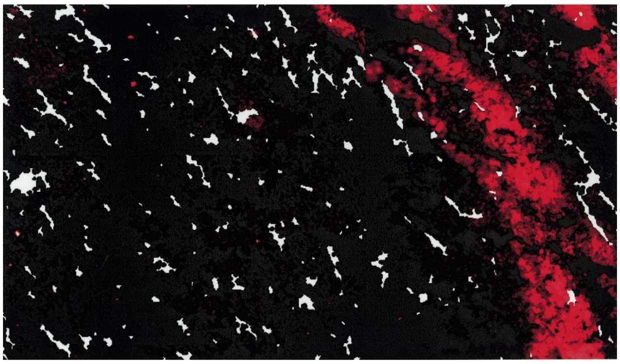

**Figure 5** Enhanced image of the PECAM-1/CD31 staining shown in Figure 3 (superimposed over the EF5/Cy3 image), showing that blood vessels (shown as white areas) are located in the brightest stained hypoxic regions (total vessel density = 189 vessels mm$^{-2}$). See text for details

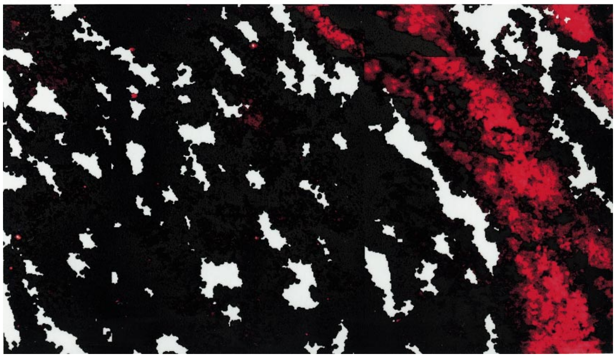

**Figure 6** Enhanced image of the Hoechst 33342 staining shown in Figure 1, showing that perfused blood vessels (shown as white areas) are generally absent in the brightest stained regions of hypoxia (superimposed over the EF5/Cy3 image). See text for details allow visualization of the relationships between hypoxia and vascular configuration. For the counting operation, vascular structures of area less than 10 μm$^2$ (corresponding to vessels less than 3.5 μm in diameter) were removed, and an area of interest (AOI) was outlined to omit artifacts and normal tissue. For each image montage, the overall area scanned and overall vascular density, as well as the X and Y spatial coordinates for each individual blood vessel, were stored on disk for later analysis.

### *H33342 image enhancement*

The H33342 stained vessels (perfused blood vessels) were similarly colour segmented, binarized and filtered. However, as the H33342 diffuses outward from the blood vessels, closely neighbouring vessels could not always be distinguished and counted automatically (Figure 6). For this reason, the enhanced PECAM-1 and H33342 images were combined using the logical 'AND' operation (in which only pixels that are 'on' in both images will be 'on' in the resulting image) to form a new image in which only those PECAM-1 vessels containing H33342 were counted. This image was again subjected to a binary closing operation to fill in the gaps between closely aligned vascular elements (Figure 7).

### *DiOC$_7$(3) image enhancement*

As an alternative to the H33342, the carbocyanine dye, DiOC$_7$(3) (Molecular Probes; 450- to 490-nm excitation, 510-nm dichroic

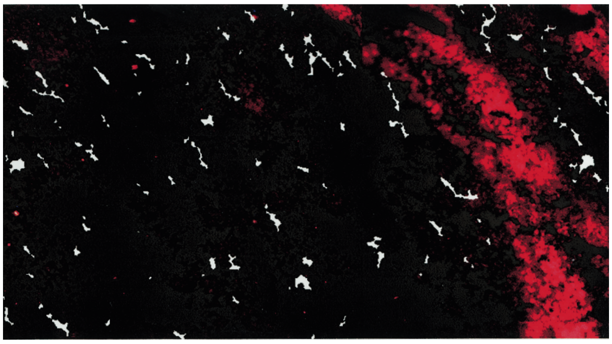

**Figure 7** Image resulting from a logical 'AND' operation on Figure 5 and 6 (superimposed over the EF5/cy3 image). As Hoechst 33342 staining does not precisely delineate vascular dimensions, this enhanced image illustrates which of the PECAM-1/CD31-stained vessels are open to flow (perfused vessel density = 95 vessels mm$^{-2}$). Regions lacking both EF5/Cy3 staining and perfused blood vessels may also correspond to intermittently closed vessels (see text for details)

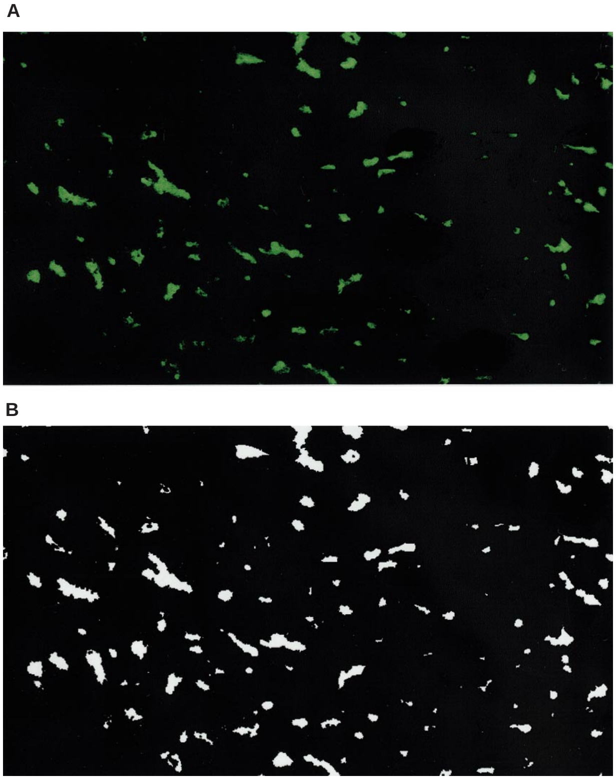

**Figure 8** (**A**) An enlarged portion of the DiOC$_7$(3) stained montage. Vessels open to flow at the time of tumour freezing are identified by brightly green-stained cells around the vessel lumens. (**B**) Enhanced image of this same field and 520-nm barrier) was also tested in a second group of tumours, as shown in Figure 8A. The DiOC$_7$(3) image was colour segmented using a predefined range of colours, and filtered twice (a 2×2 erosion and a 3×3 dilation), resulting in the enhanced image shown in Figure 8B. The DiOC$_7$(3) enhanced images have several advantages over the H33342 enhancements shown in Figure 6: (1) the colour of the DiOC$_7$(3) fluorescence varies over a very limited range, allowing a fixed colour range to be used and eliminating the manual colour segmentation; (2) the DiOC$_7$(3) stain does not

© Cancer Research Campaign 1999

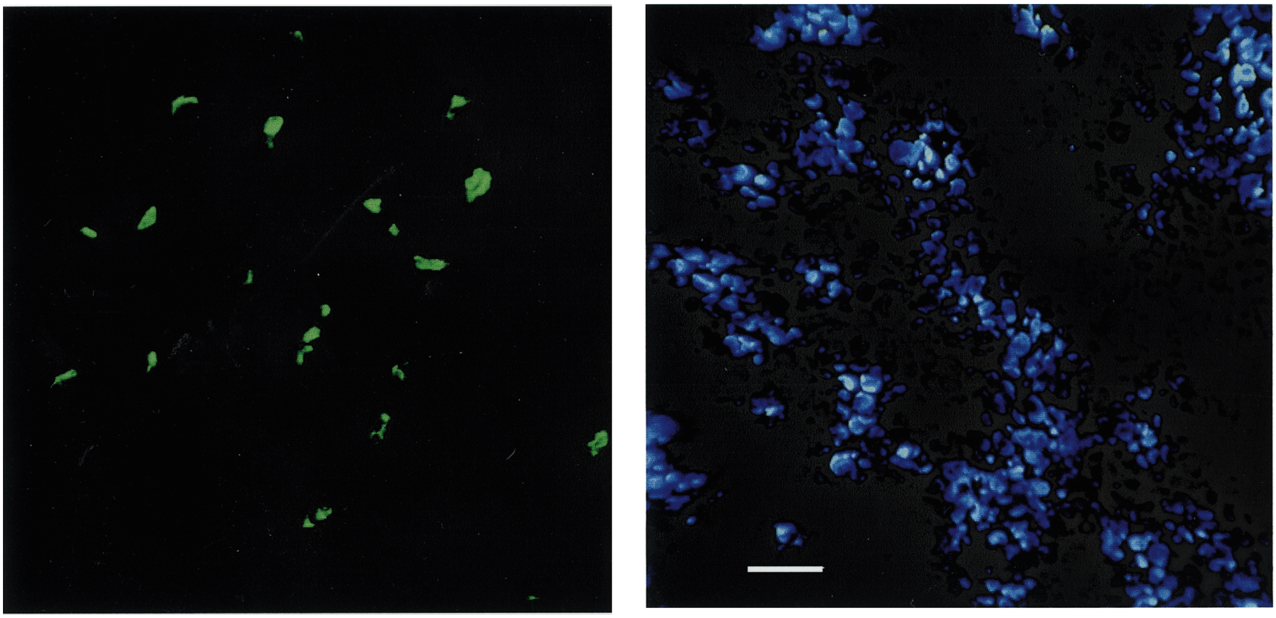

**Figure 9** Enlarged comparison of $DiOC_7(3)$ staining (on left) and H33342 staining (on right). The H33342 stain diffuses much further from the vessels than the $DiOC_7(3)$ stain, thus staining a large number of the surrounding tumour cells and producing a less precise delineation of perfused blood vessels. The two sections compare vessels of similar size, but are from different tumours. Bar = 50 µm diffuse as far from the vessels as the H33342, resulting in a much more precise count of individual vessels (Figure 9); and (3) perfused vascular density can be obtained directly, without requiring the logical addition of the $DiOC_7(3)$ and PECAM images.

### Registration of EF5 and $HbO_2$ images

Because of potential vasoactivity of the H33342 and $DiOC_7(3)$ staining, as well as the stress due to the i.v. injections, separate tumours were used to relate EF5 staining to corresponding intravascular $HbO_2$ determinations. For these studies, a 10 × image (6×6 montage) of the EF5 hypoxic marker distribution was selected to encompass the four pin-hole reference points in the section. This 10× montage was spatially aligned with the corresponding intravascular $HbO_2$ saturation map as follows. First, the $HbO_2$ data were converted to a two-dimensional bubble plot (Sigmaplot, Version 3.03, Jandel Scientific), in which the red intensities of the individual points were proportional to the individual $HbO_2$ measurements. The coordinates of the four pin-hole reference points were also included on this plot as green bubbles. This image was captured (HiJaak Graphics Suite 95) and opened in Image Pro. Using the reference pin-hole locations in both the EF5 and the $HbO_2$ images, and the Image Pro registration tool, the $HbO_2$ image was translated, rotated and scaled to match the EF5 image. Next, an AOI was defined to encompass all of the $HbO_2$ measurements, and the following information was saved on disk: (1) the X and Y coordinates of each vessel; (2) the red intensity of each vessel (which was later converted back to $HbO_2$ saturations); and (3) the red intensity of the EF5 image at locations corresponding to each blood vessel (these intensity measures encompass an approximately 25-µm-diameter sampling area). A 50-µm rectangular sampling grid was also superimposed over the EF5 image, and red intensities were recorded at each of the sampling coordinates.

### Closest-individual method

Instead of relying solely on vascular density measurements, the 'closet-individual method' of Kayar (Kayar et al, 1982; Fenton and Way, 1993) was also used to quantify diffusion distances. This analysis is much better suited to image analysis techniques, as it does not depend on distinguishing whether closely adjacent vascular elements arise from two separate vessels or from two discontinuous segments of the same vessel. A rectangular matrix of sampling points (50 µm spacing) is first computer superimposed over the selected tumour montage. Next, the distance from each sampling point to the nearest blood vessel is determined. Finally, the cumulative frequency distribution of these distances provides an estimate of the probability of encountering vessels within any specified distance from the tumour cells.

Figure 10 presents the cumulative frequency distributions for each of the three vascular stains. For each tumour, either H33342 (open triangles) or $DiOC_7(3)$ (filled triangles) stain was injected before tumour freezing and PECAM-1/CD31 staining was performed for each (open and filled circles respectively) after the fluorescent imaging. Image acquisition took approximately 20 min per montage, and image analysis required an additional 5 min. Cumulative frequency curves for total blood vessels were not different between the two groups of tumours. While the $DiOC_7(3)$ distribution is somewhat shifted to the left in relation to the H33342, this shift was also not significant. To evaluate the reproducibility of the colour segmentation procedures for each stain, four tumour montages of each stain were analysed four separate times to gauge the influence of the manual colour selections (using a different manually determined colour segmentation range each time). Overall distributions were not substantially altered by the specific colour segmentation scheme chosen and, in each case, were within the standard errors of the curves of Figure 10 (data not

© Cancer Research Campaign 1999

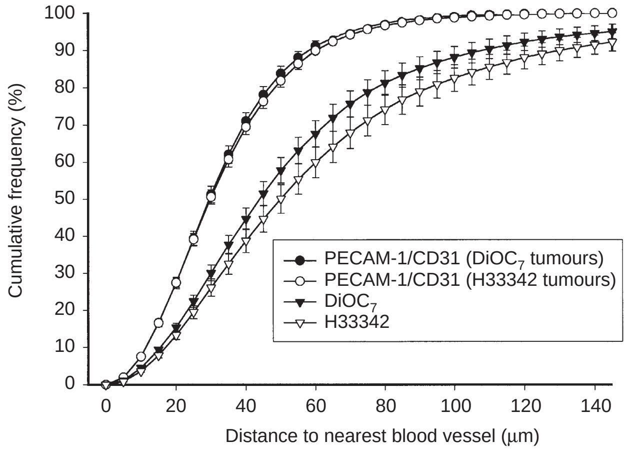

**Figure 10** Cumulative frequency distributions vs distance to the nearest blood vessel, for PECAM-1/CD31 (circles), H33342 (open triangles) and $DiOC_7(3)$ (filled triangles) staining (mean ± s.e.). Filled circles are the PECAM-1/CD31 measurements from the tumours injected with $DiOC_7(3)$, while open circles are the PECAM-1/CD31 measurements from tumours injected with the H33342. The H33342 curves are based on 21 photomontages from seven tumours, and the $DiOC_7(3)$ curves are based on 16 photomontages taken from six tumours. See text for details shown). Variations in the overall curves, as a result of analysis by different observers, were also within the standard errors of each curve (data not shown).

Based on the PECAM-1 staining, 95% of the tumour cells were within 70 μm of the nearest anatomical vessel (Figure 10). This distance cut-off provides a very rough guideline for the distance oxygen might be expected to diffuse from a well-oxygenated vessel (Vaupel, 1993) and indicates that these tumours would be fairly well oxygenated if all of the blood vessels contained sufficient oxygen. Based on the $DiOC_7(3)$ staining, however, only 76% of the tumour cells were within 70 μm of the nearest perfused vessel. If H33342 staining, rather than $DiOC_7(3)$, was used to identify perfused vessels, only 68% of the tumour cells were estimated to be within 70 μm of the nearest perfused vessel. Although not significantly different, $DiOC_7(3)$ is clearly a superior indicator of the number of perfused vessels in these tumours. The resolution is improved as a result of: (1) a higher fluorescent intensity for the $DiOC_7(3)$; (2) less photo-bleaching; and (3) an increase in the contrast between vessels and background (see Figure 9).

Figure 11 presents an example of a three-dimensional mesh plot of the relationships among EF5/Cy3 fluorescent intensity, intravascular $HbO_2$ saturation and distance to the blood vessel. For this analysis, a 50-μm rectangular grid was first computer-superimposed over each EF5 image montage (with corresponding $HbO_2$ map). At each grid point, the EF5 intensity was recorded, as well as the distance to the highest $HbO_2$ vessel located within a 150-μm radius of the grid point, and the $HbO_2$ saturation of this vessel. This analysis only includes the coordinates of those vessels that were selected for $HbO_2$ determination, i.e. greater than 8.0 μm diameter, and data from six KHT tumours were combined for the plot of Figure 11. EF5 intensity peaks correspond to areas of the tumour that were sufficiently hypoxic to bind the EF5 during the 1 h before tumour freezing. If blood vessels intermittently opened and closed during this period, EF5 binding would be reduced, and $HbO_2$ levels

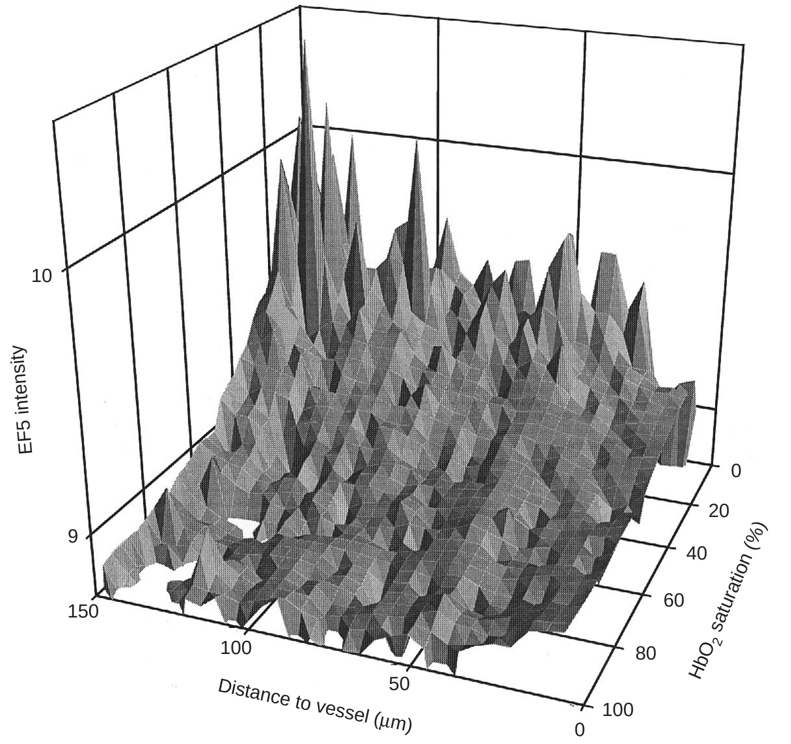

**Figure 11** Mesh plot of EF5 intensity vs distance to the nearest vessel vs intravascular $HbO_2$ saturation. Data obtained from six untreated KHT tumours are combined in this plot. $HbO_2$ saturations are measured in the tumour block adjacent to the EF5/Cy3 section, and peaks correspond to hypoxic regions in the tumour. Overall, hypoxia tends to increase with decreasing $HbO_2$ saturation and increasing distance to the nearest vessel (see text for details)

© Cancer Research Campaign 1999

at the time of freezing could be either high or low, resulting, at times, in areas with both low $HbO_2$ levels and low EF5 uptake. In general, however, the most hypoxic regions of the tumour (as predicted by the highest EF5 binding) are seen at the lowest $HbO_2$ levels and at the furthest distances from the blood vessels.

### DISCUSSION

Previous investigators have presented a variety of methods for quantitating distributions of anatomical and functional tumour microvessels, perhaps the most sophisticated being the techniques of Rijken et al (1995) and van Geel et al (1996), who also related total and perfused vascular areas to corresponding hypoxic areas. The current study not only uses a different hypoxic marker (which we believe to be an analogue variable rather than a binary segmentation), but also differs from previous methods in several important respects. (1) By using higher magnification, combined with colour segmentation procedures, rather than a grey level threshold, a more detailed image of individual blood vessels was obtained. (2) Instead of relative vascular area, the 'closest individual vessel' analysis was performed to estimate the probability of encountering blood vessels within any specified distance from the tumour cells. (3) $DiOC_7(3)$ staining was substituted for the previous H33342, resulting in a reduction in manual operator input and an increase in resolution. (4) Intravascular $HbO_2$ saturations were measured in sections adjacent to the thin sections used for the vascular and hypoxic marker staining, thus providing a quantitative validation of the hypoxic marker intensities in situ and allowing the correlation of vascular configuration with oxygen availability.

To identify anatomical blood vessels, the choice of a suitable endothelial cell marker was a critical first step. As discussed by Fox *et al* (1995), previous studies have used antibodies directed against Factor VIII-related antigen (or CD34), vimentin, lectin, alkaline phosphatase and type IV collagen, all of which suffer from low specificity and sensitivity. We used a monoclonal antibody to CD-31 (PECAM-1), in combination with the AEC chromagen, to obtain sensitive and reliable microvessel staining in frozen tissue sections. Instead of selecting a grey level threshold to delineate vascular elements, we used a highly detailed, true colour image, stored colour ranges and interactive colour selection to distinguish blood vessels from artifacts. This colour segmentation was highly reproducible, both for repeated counts by one observer and when comparing counts by different observers.

Although tumour vascularity has most often been quantified in terms of either capillary density or intercapillary distance, neither provides average diffusion distance. In addition, the maximal distance over which oxygen or drugs must diffuse to reach all regions of the tumour is even more critical than average distance in terms of both radio- and chemotherapy (Fenton and Way, 1993). Capillary density also provides no information as to the uniformity of the capillary spacing, which could in itself produce marked gradients in oxygen delivery capacity (Loats et al, 1978). The current measurements of vessel–tumour cell distances permit the determination of both the median and the maximal distances that oxygen and nutrients must diffuse to reach all points throughout the tumour, as well as the entire distribution of these distances.

To quantify perfused blood vessels, previous studies have generally relied on H33342 staining. As shown in the current results, $DiOC_7(3)$ staining offers several key advantages over the H33342. First, H33342 diffuses much further from the blood vessels in 1 min than does $DiOC_7(3)$ in 4 min. As shown in Figure 9, this results in substantial overlap among the H33342-stained vessels. Subsequent image processing is therefore much more difficult, and the overlap between PECAM-1 and H33342 stained vessels must be determined to estimate vascular density. With the $DiOC_7(3)$ (Figure 9), vascular elements are: (1) more clearly defined, (2) much brighter in contrast and intensity and (3) characterized by a more limited range of colours. This allows an automatic colour segmentation to be used for multiple $DiOC_7(3)$ images, rather than requiring a separate manual segmentation for each image, as required for the H33342 images. Finally, because the $DiOC_7(3)$ vessels are more clearly defined, vascular density counts can be performed directly on the $DiOC_7(3)$ images, rather than by measuring the overlap between $DiOC_7(3)$ and PECAM-1.

The current methods allow a comprehensive, microregional mapping of heterogeneities in three inter-related physiological parameters: (1) total and perfused vascular structure, (2) intravascular oxygen availability and (3) hypoxic development. Although this detailed information is limited to one time point per tumour, results obtained from matched-volume tumours can be combined to define the physiological changes that accompany tumour growth, local irradiation or various manipulations of blood flow and oxygen delivery. Finally, by evaluating the changing relationships among EF5/Cy3 intensity, $HbO_2$ saturation and distance from the vessel, a quantitative estimate of changes in oxygen consumption between tumours is also possible.

### ACKNOWLEDGEMENTS

The authors would like to thank Dr Thomas EJ Gayeski, Departments of Anesthesiology and Physiology & Pharmacology, University of Rochester Medical Center, for the use of his laboratory and cryospectrophotometer. Financial support was provided by NIH grants CA52586 and CA28332.

### REFERENCES

Brown JM and Giaccia AJ (1994) Tumour hypoxia: the picture has changed in the 1990s. (Review). *Int J Radiat Biol* **65**: 95–102

Fenton BM and Gayeski TEJ (1990) Determination of microvascular oxyhemoglobin saturations using cryospectrophotometry. *Am J Physiol* **259**: H1912–H1920

Fenton BM and Way BW (1993) Vascular morphometry of KHT and RIF-1 murine sarcomas. *Radiother Oncol* **28**: 57–62

Fox SB, Leek RD, Weekes MP, Whitehouse RM, Gatter KC and Harris AL (1995) Quantitation and prognostic value of breast cancer angiogenesis: comparison of microvessel density, Chalkley count, and computer image analysis. *J Pathol* **177**: 275–283

Horsman MR, Nordsmark M, Khalil AA, Hill SA, Chaplin DJ, Siemann DW and Overgaard J (1994) Reducing acute and chronic hypoxia in tumours by combining nicotinamide with carbogen breathing. *Acta Oncologica* **33**: 371–376

Huang X, Molema G, King S, Watkins L, Edgington TS and Thorpe PE (1997) Tumor infarction in mice by antibody-directed targeting of tissue factor to tumour vasculature. *Science* **275**: 547–550

Kayar SR, Archer PG, Lechner AJ and Banchero N (1982) The closest-individual method in the analysis of the distribution of capillaries. *Microvasc Res* **24**: 326–341

Klauber N, Parangi S, Flynn E, Hamel E and Damato RJ (1997) Inhibition of angiogenesis and breast cancer in mice by the microtubule inhibitors 2-methoxyestradiol and taxol. *Cancer Res* **57**: 81–86

Loats JT, Sillau AH and Banchero N (1978) How to quantify skeletal muscle capillarity. *Adv Exp Med Biol* **94**: 41–48

Lord EM, Harwell L and Koch CJ (1993) Detection of hypoxic cells by monoclonal antibody recognizing 2-nitroimidazole adducts. *Cancer Res* **53**: 5721–5726

Olive PL and Durand RE (1987) Characterization of a carbocyanine derivative as a fluorescent penetration probe. *Cytometry* **8**: 571–575

© Cancer Research Campaign 1999

O'Reilly MS, Boehm T, Shing Y, Fukai N, Vasios G, Lane WS, Flynn E, Birkhead JR, Olsen BR and Folkman J (1997) Endostatin: an endogenous inhibitor of angiogenesis and tumor growth. *Cell* **88**: 277–285

Rijken PFJW, Bernsen HJJA and van der Kogel AJ (1995) Application of an image analysis system to the quantitation of tumor perfusion and vascularity in human glioma xenografts. *Microvasc Res* **50**: 141–153

Smith KA, Hill SA, Begg AC and Denekamp J (1988) Validation of the fluorescent dye Hoechst 33342 as a vascular space marker in tumours. *Br J Cancer* **57**: 247–253

Thomson JE and Rauth AM (1974) An in vitro assay to measure the viability of KHT tumor cells not previously exposed to culture conditions. *Radiat Res* **58**: 262–276

Trotter MJ, Chaplin DJ, Durand RE and Olive PL (1989*a*) The use of fluorescent probes to identify regions of transient perfusion in murine tumors. *Int J Radiat Oncol Biol Phys* **16**: 931–934

Trotter MJ, Chaplin DJ and Olive PL (1989*b*) Use of a carbocyanine dye as a marker of functional vasculature in murine tumours. *Br J Cancer* **59**: 706–709

van Geel IPJ, Oppelaar H, Rijken PFJW, Bernsen HJJA, Hagemeier NEM, van der Kogel AJ, Hodgkiss RJ and Stewart FA (1996) Vascular perfusion and hypoxic areas in RIF-1 tumours after photodynamic therapy. *Br J Cancer* **73**: 288–293

Vaupel PW (1993) Oxygenation of solid tumors. In *Drug Resistance in Oncology*, Teicher BA. (ed), pp. 53–85. Marcel Dekker: New York

Vecchi A, Garlanda C, Lampugnani MG, Resnati M, Matteucci C, Stoppacciaro A, Schnurch H, Risau W, Ruco L, Mantovani A and Dejana E (1994) Monoclonal antibodies specific for endothelial cells of mouse blood vessels. Their application in the identification of adult and embryonic endothelium. *Eur J Cell Biol* **63**: 247–254

© Cancer Research Campaign 1999

## References

[PDF_00633] Fox S. B., Leek R. D., Weekes M. P., Whitehouse R. M., Gatter K. C., Harris A. L. (1995). Quantitation and prognostic value of breast cancer angiogenesis: comparison of microvessel density, Chalkley count, and computer image analysis.. J Pathol.

[PDF_00637] Horsman M. R., Nordsmark M., Khalil A. A., Hill S. A., Chaplin D. J., Siemann D. W., Overgaard J. (1994). Reducing acute and chronic hypoxia in tumours by combining nicotinamide with carbogen breathing.. Acta Oncol.

[PDF_00641] Huang X., Molema G., King S., Watkins L., Edgington T. S., Thorpe P. E. (1997). Tumor infarction in mice by antibody-directed targeting of tissue factor to tumor vasculature.. Science.

[PDF_00644] Kayar S. R., Archer P. G., Lechner A. J., Banchero N. (1982). The closest-individual method in the analysis of the distribution of capillaries.. Microvasc Res.

[PDF_00652] Lord E. M., Harwell L., Koch C. J. (1993). Detection of hypoxic cells by monoclonal antibody recognizing 2-nitroimidazole adducts.. Cancer Res.

[PDF_00658] O'Reilly M. S., Boehm T., Shing Y., Fukai N., Vasios G., Lane W. S., Flynn E., Birkhead J. R., Olsen B. R., Folkman J. (1997). Endostatin: an endogenous inhibitor of angiogenesis and tumor growth.. Cell.

[PDF_00654] Olive P. L., Durand R. E. (1987). Characterization of a carbocyanine derivative as a fluorescent penetration probe.. Cytometry.

[PDF_00661] Rijken P. F., Bernsen H. J., van der Kogel A. J. (1995). Application of an image analysis system to the quantitation of tumor perfusion and vascularity in human glioma xenografts.. Microvasc Res.

[PDF_00664] Smith K. A., Hill S. A., Begg A. C., Denekamp J. (1988). Validation of the fluorescent dye Hoechst 33342 as a vascular space marker in tumours.. Br J Cancer.

[PDF_00667] Thomson J. E., Rauth A. M. (1974). An in vitro assay to measure the viability of KHT tumor cells not previously exposed to culture conditions.. Radiat Res.

[PDF_00670] Trotter M. J., Chaplin D. J., Durand R. E., Olive P. L. (1989). The use of fluorescent probes to identify regions of transient perfusion in murine tumors.. Int J Radiat Oncol Biol Phys.

[PDF_00673] Trotter M. J., Chaplin D. J., Olive P. L. (1989). Use of a carbocyanine dye as a marker of functional vasculature in murine tumours.. Br J Cancer.

[PDF_00682] Vecchi A., Garlanda C., Lampugnani M. G., Resnati M., Matteucci C., Stoppacciaro A., Schnurch H., Risau W., Ruco L., Mantovani A. (1994). Monoclonal antibodies specific for endothelial cells of mouse blood vessels. Their application in the identification of adult and embryonic endothelium.. Eur J Cell Biol.

[PDF_00676] van Geel I. P., Oppelaar H., Rijken P. F., Bernsen H. J., Hagemeier N. E., van der Kogel A. J., Hodgkiss R. J., Stewart F. A. (1996). Vascular perfusion and hypoxic areas in RIF-1 tumours after photodynamic therapy.. Br J Cancer.

